# Prediction of neovascular age-related macular degeneration recurrence using optical coherence tomography images with a deep neural network

**DOI:** 10.1038/s41598-024-56309-6

**Published:** 2024-03-11

**Authors:** Juho Jung, Jinyoung Han, Jeong Mo Han, Junseo Ko, Jeewoo Yoon, Joon Seo Hwang, Ji In Park, Gyudeok Hwang, Jae Ho Jung, Daniel Duck-Jin Hwang

**Affiliations:** 1 Department of Applied Artificial Intelligence, Sungkyunkwan University, Seoul, Korea; 2 Department of Human-Artificial Intelligence Interaction, Sungkyunkwan University, Seoul, Korea; 3 Department of Ophthalmology, Seoul National University College of Medicine, Seoul, Korea; 4 Kong Eye Center, Seoul, Korea; 5 Raondata, Seoul, Korea; 6 Seoul Plus Eye Clinic, Seoul, Korea; 7 Department of Medicine, Kangwon National University Hospital, Kangwon National University School of Medicine, Chuncheon, Gangwon-do Korea; 8 Department of Ophthalmology, Hangil Eye Hospital, 35 Bupyeong-Daero, Bupyeong-gu, Incheon, 21388 Korea; 9 Department of Ophthalmology, Catholic Kwandong University College of Medicine, Incheon, Korea; 10 Lux Mind, Incheon, Korea

**Keywords:** Machine learning, Retinal diseases, Medical imaging

## Abstract

Neovascular age-related macular degeneration (nAMD) can result in blindness if left untreated, and patients often require repeated anti-vascular endothelial growth factor injections. Although, the treat-and-extend method is becoming popular to reduce vision loss attributed to recurrence, it may pose a risk of overtreatment. This study aimed to develop a deep learning model based on DenseNet201 to predict nAMD recurrence within 3 months after confirming dry-up 1 month following three loading injections in treatment-naïve patients. A dataset of 1076 spectral domain optical coherence tomography (OCT) images from 269 patients diagnosed with nAMD was used. The performance of the model was compared with that of 6 ophthalmologists, using 100 randomly selected samples. The DenseNet201-based model achieved 53.0% accuracy in predicting nAMD recurrence using a single pre-injection image and 60.2% accuracy after viewing all the images immediately after the 1st, 2nd, and 3rd injections. The model outperformed experienced ophthalmologists, with an average accuracy of 52.17% using a single pre-injection image and 53.3% after examining four images before and after three loading injections. In conclusion, the artificial intelligence model demonstrated a promising ability to predict nAMD recurrence using OCT images and outperformed experienced ophthalmologists. These findings suggest that deep learning models can assist in nAMD recurrence prediction, thus improving patient outcomes and optimizing treatment strategies.

## Introduction

Age-related macular degeneration (AMD) can be classified into dry and wet types; wet AMD, that is neovascular AMD (nAMD), involves the growth of new blood vessels in the subretinal or intraretinal layers, resulting in hemorrhage, edema, and ultimately blindness if left untreated^1,2^. The standard treatment for nAMD involves intravitreal injections of anti-vascular endothelial growth factor (VEGF) agents to suppress and regress these new blood vessels^3^. Patients receiving AMD treatment often require repeated injections, and various treatment protocols, such as fixed^4–7^, pro re nata (PRN)^8–10^, and treat-and-extend (T&E)^11^, are employed according to the retreatment modality. Typically, the next treatment course is decided after three loading injections, with a growing trend towards using the T&E method to reduce vision loss owing to recurrence (ASRS survey)^12^. However, T&E may pose the risk of overtreatment based on the patient's condition^13^. Limited research exists on the type of patients who could have their injection intervals extended or monitored without receiving injections.

With the advancement of artificial intelligence (AI), numerous research findings have been reported on the diagnosis and treatment of retinal diseases. A model predicting whether the injection interval would be less than 5 weeks (high treatment burden) or more than 10 weeks (low treatment burden) when administering T&E using anti-VEGF agents for nAMD treatment has been reported^14,15^. Predicting the patients requiring immediate T&E treatment (owing to recurrence within 3 months after the three loading injections for nAMD), those with a longer T&E interval, and those considering PRN treatment after 3 months could aid in treatment planning. Thus, we aimed to develop an AI model to predict the group of nAMD-naïve patients who would experience recurrence within 3 months after confirming dry-up 1 month after three loading injections. Additionally, we performed experiments to compare the predictive accuracy of this model with that of ophthalmologists and retinal specialists.

## Results

In this study, we conducted a study based on 1076 OCT images from 269 participants. The mean ages were 70.70 ± 8.84 years. Detailed information on the data used in this study is presented in Table 1.

**Table 1:** Baseline characteristics.

Variables	Neovascular AMD (N = 269)
Age, years (IQR)	70.7 (64–77, median 71)
Sex, n (%)	
Male	155 (58)
Female	114 (42)
Eye treated, n (%)	
Right	141 (52)
Left	128 (48)
Underlying disease, n (%)	
Hypertension	138 (51)
Diabetes	65 (24)

*AMD* age-related macular degeneration, *IQR* interquartile range.

### Model performance

As shown in Table 2, the proposed model based on DenseNet201 achieved an accuracy of 53.0% in predicting recurrence after viewing only one pre-injection image and 60.2% accuracy in predicting recurrence after seeing all the images immediately after the 1st, 2nd, and 3rd injections. Table 2 summarizes the comparison of the performances of our model with baseline models upon predicting recurrence after viewing all the four images. Our model demonstrated the highest accuracy, followed by InceptionV3 (58.8%) and DenseNet169 (59.7%). In addition, Table 3 shows that both LSTM and attention module demonstrated the highest performance (60.22%) on combining sequential features of multiple images. We employed the same DenseNet201 encoder for all the fusion methods and the same data conditions were applied to all the models.

**Table 2:** Comparison of the performance among baseline encoders.

Method	Accuracy
VGG16	0.5413
Xception	0.5502
ResNet50	0.5506
InceptionV3	0.5876
DenseNet121	0.4591
DenseNet169	0.5966
DenseNet201	0.6020

All the models obtained the following four images as one data input: an initial image, spectral domain optical coherence tomography (SD-OCT) after the first injection, SD-OCT after the second injection, and SD-OCT after the third injection. The same fusion method, namely, attention, was employed.

**Table 3:** Performance of the fusion baselines.

Fusion	Accuracy	Sensitivity	Specificity	F1 Score
LSTM^a^	0.6022	0.6058	0.5985	0.6021
Concat^b^	0.5799	0.6386	0.5538	0.5649
Attention	0.6022	0.6160	0.5903	0.6019
Average	0.5874	0.7273	0.5514	0.5495

The same DenseNet201 encoder was used for each fusion baseline, and the performance was evaluated on a multi-instance task that used four input images, including a pre-injection image and three loading injection images.

^a^LSTM and ^b^Concat denote Long-Short Term Memory and Concatenation, respectively.

We conducted an ablation study to examine the effects of clinical data (sex, age, diabetes, and high blood pressure) on nAMD recurrence prediction. The clinical data were preprocessed using one-hot encoding and inserted as input values along with the OCT images in the model. As shown in Table 4, the accuracy of the model was 59.04% and 60.22% with and without clinical data, respectively, indicating that the inclusion of clinical data did not significantly enhance the performance of the model. We also assess the impact of the type of anti-VEGF agent used on enhancing prediction performance. Table 4 also illustrates that the choice of anti-VEGF treatment for each patient does not influence the recurrence results. It is crucial to emphasize that, since this experiment solely aims to predict whether recurrence will occur within 3 months from the last injection, any association with recurrence beyond this timeframe is not analyzed. Therefore, the findings indicate that incorporating the patient's clinical information did not enhance the model's ability to predict nAMD recurrence within the first 3 months following the last injection.

**Table 4:** Ablation study on numeric clinical data.

	Accuracy	Sensitivity	Specificity	F1 Score
OCT only	0.6022	0.6058	0.5985	0.6021
OCT with Clinical Data	0.5904	0.6133	0.5441	0.5790
OCT with Clinical Data and the type of anti-VEGF agent	0.5928	0.6182	0.5962	0.5815

Clinical Data include values for age, sex, diabetes, and high blood pressure.

### Performance comparison with ophthalmologists

Table 5 presents the predictions of our proposed model and those of the six ophthalmologists concerning disease recurrence after examining a single pre-injection image. The ophthalmologists’ prediction accuracy ranged from 49 to 56%, with an average accuracy of 52.17%, whereas the accuracy of our proposed model was 53.0%, which was higher than the average accuracy of ophthalmologists. Note that, both the ophthalmologists and the model were not exposed to the other three post-injection images during the single pre-injection image task. Therefore, the results demonstrated that our model can predict recurrence more accurately than experienced ophthalmologists upon analyzing a single pre-injection image.

**Table 5:** Comparison of the performances between the model and human experts on the pre-injection image-only task.

	Input	Accuracy	Sensitivity	Specificity	F1 score	Precision
F1^a^	A	0.5500	0.6078	0.4898	0.5794	0.5536
F2^a^	A	0.5600	0.3137	0.8163	0.4211	0.6400
F3^a^	A	0.5100	0.5882	0.4286	0.5505	0.5172
R1^b^	A	0.5100	0.3529	0.6735	0.4235	0.5294
R2^b^	A	0.5100	0.6078	0.4082	0.5586	0.5167
RS^c^	A	0.4900	0.3529	0.6327	0.4138	0.5000
Average of Doctors	A	0.5217	0.4706	0.5749	0.4912	0.5428
Model	A	0.5300	0.6667	0.3878	0.5913	0.5313

Input A denotes a single pre-injection image.

^a^F1, F2, and F3 denote retinal fellows with 2 years of experience as a retina specialist.

^b^R1 and R2 denote ophthalmology residents with 1 and 3 years of experience, respectively.

^c^RS refers to retina specialist with more than 10 years of clinical experience.

Furthermore, we conducted extensive experiments by providing the model and ophthalmologists with four images, (comprising pre-injection and immediately after each of the three loading injections) per patient. As shown in Table 6, when all the four images were provided, the ophthalmologists' accuracy ranged from 51 to 56%, with an average accuracy of 53.3%, whereas the model achieved an accuracy of 60.2%. This indicates that ophthalmologists did not demonstrate a significant improvement in the accuracy despite examining more images, whereas the model demonstrated a 7% improvement in accuracy.

**Table 6:** Comparison of the performances between the model and human experts on pre-injection image and all images immediately after each of the three injections.

	Input	Accuracy	Sensitivity	Specificity	F1score	Precision
F1^a^	A + B + C + D	0.5500	0.3529	0.7551	0.4444	0.6000
F2^a^	A + B + C + D	0.5300	0.3333	0.7347	0.4198	0.5667
F3^a^	A + B + C + D	0.5600	0.4902	0.6327	0.5319	0.5814
R1^b^	A + B + C + D	0.5100	0.2941	0.7347	0.3797	0.5357
R2^b^	A + B + C + D	0.5400	0.6078	0.4694	0.5741	0.5439
RS^c^	A + B + C + D	0.5100	0.3137	0.7143	0.3951	0.5333
Doctor Average	A + B + C + D	0.5333	0.3987	0.6735	0.4575	0.5602
Model	A + B + C + D	0.6022	0.6275	0.5510	0.6095	0.5926

A, B, C, and D denote the pre-injection image, optical coherence tomography (OCT) image after the first injection, OCT image after the second injection, and OCT image after the third injection, respectively.

^a^F1, F2, and F3 denote retinal fellows with 2 years of experience as a retina specialist.

^b^R1 and R2 denote ophthalmology residents with 1 and 3 years of experience, respectively.

^c^RS refers to retina specialist with more than 10 years of experience.

**Figure 1:**
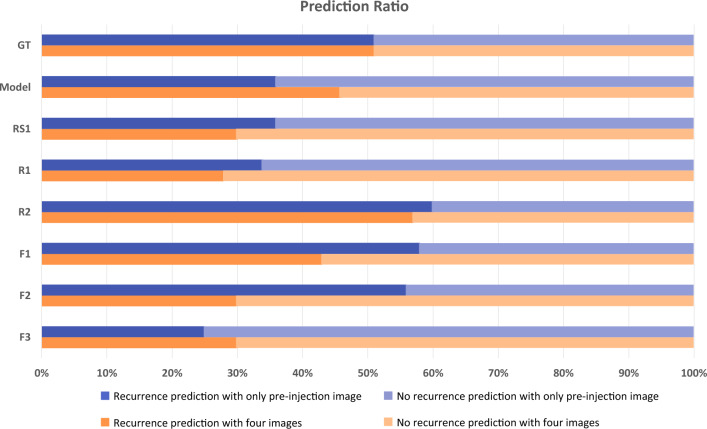
The prediction ratio of the proposed model and retinal professionals. The prediction ratio refers to the ratio between recurrence predictions and non-recurrence predictions. The blue bar represents the prediction ratio between recurrence and non-recurrence for the pre-injection image-only task, while the orange bar represents the prediction ratio between recurrence and non-recurrence for the pre-injection image and the three images immediately after each of the three injections. *F* retina fellow, *R* ophthalmology resident, *RS* retina specialist, *GT* ground truth.

To investigate whether the basic knowledge of ophthalmologists biased their predictions when considering more images, we calculated the change in the ratios of predictions between the model and doctors when examining a single image and four images. Figure 1 depicts the change in the ratio in the recurrence prediction values of the model and six ophthalmologists when one and four images were shown. Among the six ophthalmologists, all except “F2” changed their predicted value from "recurrence" to "no recurrence" when the scans immediately after the three-loading injections were additionally shown, whereas the model's predictions were consistent with the actual ground truth. This suggested that experienced ophthalmologists altered their predictions based on their background knowledge and experience when presented with additional imaging information, whereas our model considered only the information in the OCT image to generate accurate predictions.

The Fleiss' kappa coefficient was used to measure the level of agreement between all the ophthalmologists and the proposed model in predicting the recurrence of nAMD using both a single-input image task with only the pre-injection image and a multi-image input task. The results demonstrated poor agreement among the ophthalmologists, with coefficients of 0.0659 and 0.0380 for the single- and multi-input image tasks, respectively (P < 0.001). In addition, poor agreement was observed between the proposed model and ophthalmologists, with coefficients of 0.0727 and 0.0463 in the single-and multi-image input tasks, respectively (P < 0.002). Statistical analysis revealed that the level of agreement between the ophthalmologists and models was lower in the multi-image input task than in the single-image input task.

### Grad-CAM visualization

**Figure 2:**
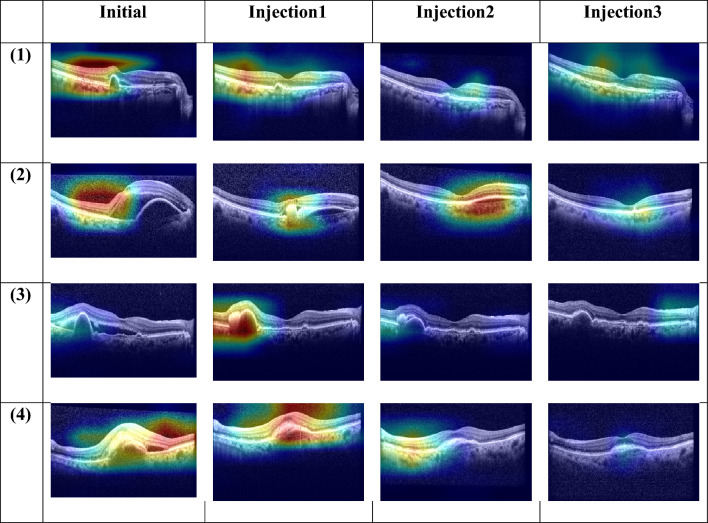
Heat maps generated using gradient weighted class activation map (Grad-CAM) for the model’s prediction on neovascular age-related macular degeneration (nAMD) recurrence. Grad-CAM visualized the pathologic regions most closely associated with the recurrence of nAMD on the four optical coherence tomography (OCT) images: a pre-injection image, OCT after first injection, OCT after second injection, and OCT after third injection.

In Fig. 2, gradient-weighted class activation mapping (Grad-CAM) produced heat maps that highlight the regions typically considered by retinal professionals for predicting recurrence in nAMD on OCT images. The representative heat maps demonstrate that our proposed model followed a similar approach to that of human experts when assessing OCT images.

## Discussion

Our study's aim was to develop a sophisticated deep learning model capable of predicting the likelihood of future nAMD recurrence and assess its predictive accuracy. Remarkably, our model achieved substantial success in predicting nAMD recurrence within the next 3 months based on the follow-up time 1 month after the 3rd injection, exhibiting a performance level on par with or surpassing that of seasoned retinal specialists. Additionally, our study demonstrated that clinical variables, including age, sex, diabetes, and high blood pressure, did not exert a considerable effect on nAMD recurrence within the examined 6-month timeframe.

The developed AI model was designed to predict the possibility of recurrence within the next 3 months based on the follow-up time 1 month after the last injections of three loading anti-VEGF therapy in patients with nAMD. It targeted treatment-naïve patients who underwent three loading injections and assessed the likelihood of recurrence 3 months after the evaluation of the 1-month-interval post-third injection treatment effect. We anticipated that this would aid in determining subsequent treatments following three loading injections in nAMD management, and investigated whether the model could provide additional assistance compared to a physician's judgment when considering PRN, T&E, or fixed regimen follow-up treatments. To date, AI research has primarily focused on the treatment burden^14–17^, and this is the first to predict the possibility of recurrence within the next 3 months, starting 1 month after the last injection of three loading treatments, which holds great significance. In particular, with T&E treatment, continuous injections are required even in cases with no recurrence; therefore, it is expected that the number of injections could be reduced in some cases through PRN treatment^18^; however, it is currently impossible to accurately predict the group that would benefit more from T&E or PRN. Moreover, in our study, the prediction accuracy of experienced retinal specialists (0.57) was not significantly higher than that of general ophthalmologists (0.54), indicating the difficulty in predicting recurrence beforehand. Based on the findings of our study, we anticipate that a model capable of predicting recurrence within 3 months (based on the 1 month follow up after the last treatment) more accurately than experienced retinal specialists could be helpful in clinical settings.

Our model was able to determine recurrence by analyzing four consecutive OCT images obtained during three loading injections. Previous studies predicted the treatment burden by learning from the pre-treatment OCT images and OCT images after one injection, and reported an improvement in the area under the curve (AUC) from 0.64 with pre-treatment OCT images alone to 0.69 when post-treatment OCT images were also utilized^15^. We confirmed that the prediction accuracy improved when OCT images obtained during the injection treatment process were additionally learned. Upon learning from pre-injection images alone, the accuracy was 0.53; however, it increased to 0.59 when the three injection images were also learned, a value comparable to the accuracy of retinal specialists (0.57). This difference could be because the retinal specialists considered the nAMD subtypes (i.e., PCV, RAP, and other typical AMD) when predicting recurrence based solely on pre-injection images, whereas the AI model made predictions without considering these subtypes. However, when our AI model learned from additional post-injection images, it surpassed the prediction accuracy of retinal specialists by learning the changes in features, such as subretinal fluid (SRF), pigment epithelial detachment (PED), and intraretinal fluid (IRF), that occurred during the loading injection process. In particular, it was evident that the model focused on the changes in the SRF, retinal PED, and subretinal hyperreflective material (SHRM) during injection treatment upon examining the heat map of the group accurately predicted by the AI model.

**Figure 3:**
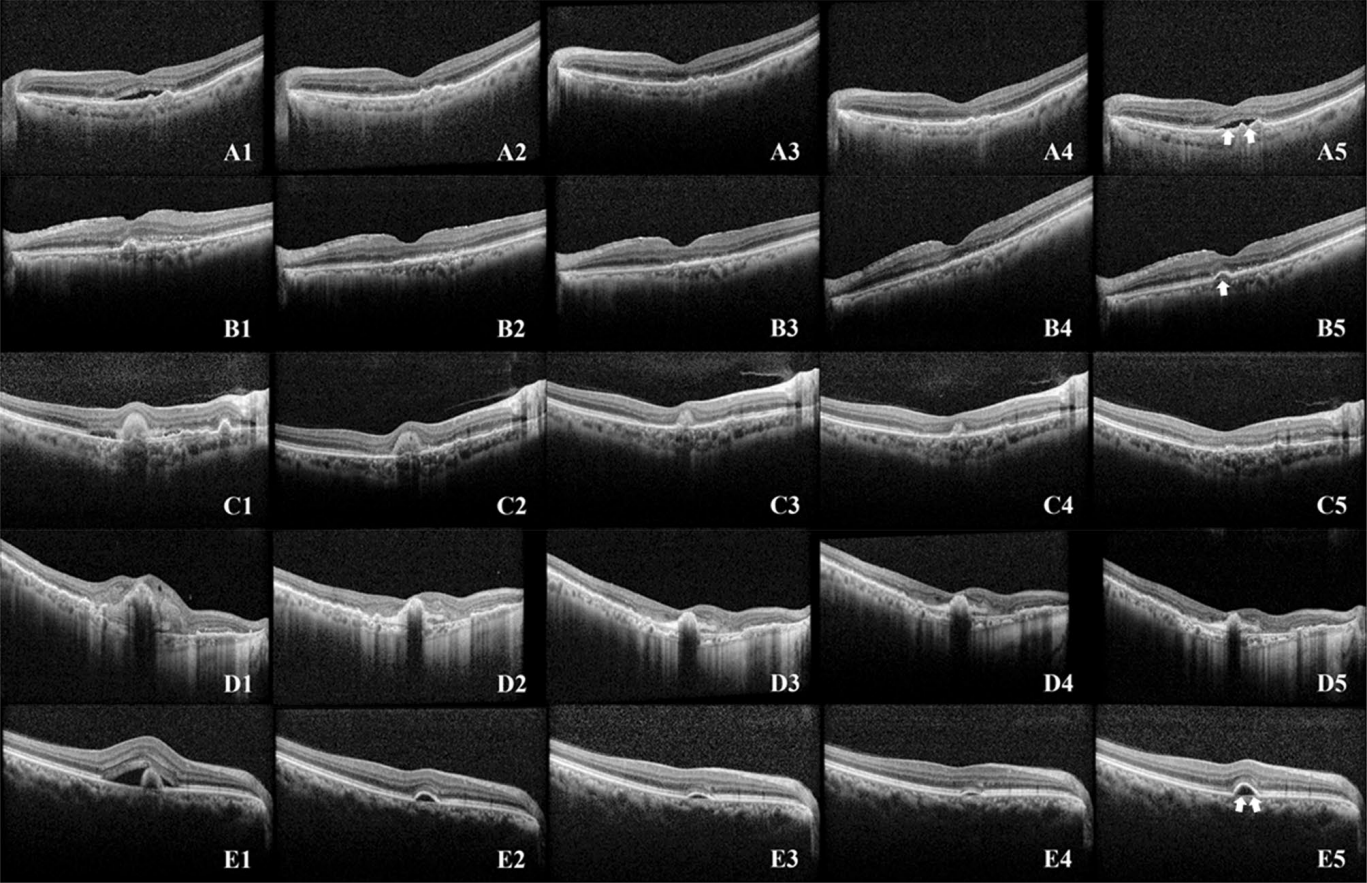
Cases that the AI model provided a false prediction. A1, B1, C1, D1, E1 were taken before first injection, A2, B2, C2, D2, E2 were taken 1 month after first injection, A3, B3, C3, D3, E3 were 1 months after second loading injections, A4, B4, C4, D4, E4 were taken 1 months after three loading injections, and A5, B5, C5, D5, E5 were taken 4 month after the three loading injections. OCT after the first injection showed a favorable outcome (A2 and B2), however within 3 months after loading injections the SRF reappeared (A5, arrow), or the PED reappeared (B5 and arrow); SHRM decreased gradually over the three loading injections, but almost all SHRM disappeared (C5), or no recurrence was observed (D5); the OCT showed only some PED after three loading injections (E4), but the increased PED proved to be a recurrence (E5); all of the above cases that the AI model wrongly predicted.

Predicting a recurrence within a 3-month timeframe following three loading injections is a challenging task. Both human specialists and AI model showed limited ability to accurately predict outcomes based just on a single pre-treatment OCT image, resulting in almost random results. However, the accuracy of the prediction showed an improvement when assessed using the OCT images taken after the first, second, and third consecutive injections. This improvement was observed not only among human experts but also in the AI model. A trend was observed that eyes that exhibit favorable response following a single injection likely to showed lower recurrence and remained as dry macula. Examining the heatmap highlighted by the AI model, it is evident that the AI focuses on areas with significant changes, such as SRF, IRF, and SHRM. This observation suggests that the AI is making accurate assessments, eliminating the need for separate learning of individual lesions (Fig. 2). However, this pattern did not apply to patients who developed a minor SRF or a small increase in PED within 3 months after loadings injections. Conversely, there was a gradual decrease in fluid over the three loading injections for some patients, and these individuals did not experience another relapse for a maximum of 3 months. Figure 3 showed the cases that the AI model provided a false prediction. To emphasize, predicting a relapse within a specific period after three loading injections is a challenging task, and to the best of our knowledge, our study is the first to attempt such a prediction.

In this study, we trained an AI model using convolutional neural network (CNN) techniques. This approach distinguishes our research from previous studies that trained models by classifying OCT images into SRF, IRF, PED, retinal photoreceptors, and other categories using autosegmentation techniques and learning their volumes^15–17^. The ability to learn using a CNN without specifying a detailed learning method highlights the strength of our study. However, difficulty in precisely determining the parts of the OCT images that the model relies upon for predicting recurrence is a possible limitation. Although we could approximately infer the areas that the model emphasized on using Grad-CAM in this study, future follow-up research may provide more specific and clear heat maps for identifying the crucial features that contribute to recurrence. Interestingly, ophthalmologists exhibited lower prediction accuracy when predicting recurrence based on the three injection treatment courses (0.50) than when they made predictions solely according to the pre-treatment images (0.54), which suggests that it may be more challenging for ophthalmologists to accurately predict recurrence when they rely on criteria, such as improved findings on OCT images or drying of lesions, assuming that these indicate a lower likelihood of recurrence.

As shown in Table 3, the LSTM and attention modules demonstrated comparable performance in predicting the nAMD recurrence in the multi-image task. This could be attributed to the fact that the pre-injection, 1st, 2nd, and 3rd injection scans are sequential data at monthly intervals, making the LSTM, which is designed for sequential data, highly effective. In addition, the attention mechanism, which highlights and focuses on information that significantly affects recurrence among the four images, also demonstrated superior performance in this multi-instance input task. This suggests that the attention module enables the deep learning model to selectively attend to the regions of an OCT image that contain the most informative features of nAMD recurrence. Despite the remarkable performance of LSTM, we selected the attention mechanism as our ultimate model fusion method to compute the attention score, representing the model's influence on recurrence prediction based on the four images. In addition, because attention can alleviate the computational burden of processing numerous images by enabling the model to concentrate solely on the most pertinent parts of each image, the attention mechanism is better suited for this multi-image prediction task. We validated the accuracy of our model’s recurrence prediction using Grad-CAM, a visualization technique that identifies critical areas in each of the four input images. We verified that our model correctly predicted recurrence by focusing on relevant regions upon analyzing the heatmap generated by Grad-CAM.

This study has several limitations. First, the variety and number of available SD-OCT images were limited. All the images were acquired using a single OCT device. In future studies, external validation using OCT devices from various manufacturers is warranted. Second, the sample size was small. To overcome this problem, we applied Leave-One-Out Cross Validation (LOOCV), a reliable technique for studies in the medical field, to small datasets^19,20^. Although the LOOCV has the benefit of increasing the validly of the training dataset by utilizing all the data for training, it is computationally intensive and more susceptible to overfitting than K-fold Cross Validation^21^. Thus, as depicted in Table 7, K-fold validations were also performed to assess the variance of the measure. The results consistently demonstrated performance trends across all folds, affirming the model's stability in responding to the dataset. Opting for LOOCV instead of traditional K-fold validation allowed us to leverage the advantages of LOOCV as a specific instance of k-fold cross-validation. This choice provided a thorough evaluation of the model's generalizability. However, since our study analyzed four OCT images, which were captured monthly, for identifying the features, the risk of overfitting was higher when the four images were used as input data. While we have implemented diverse strategies including dropout, early stopping, and regularization to mitigate this concern, we anticipate that the model's performance could further improve with larger datasets. Additionally, the choice of a 0.5 dropout rate for all layers, aimed at addressing overfitting, may also impact the generalizability of the study results. Third, this study employs a pre-trained model from the ImageNet dataset, which is trained on RGB data. The application of such a pre-trained model to grayscale medical images may result in a decline in performance. Fourth, we have not tested whether using multiple images that make up the OCT volume per time point instead of a single line scan shows better performance. If technical issues are resolved and recurrence is predicted using multiple images per time point, this model may be useful in extrafoveal CNV cases. Fifth, the nAMD subtypes could not be distinguished. The response to the injections and prognosis may vary for each nAMD subtype; we did not consider that the subtypes. A higher prediction performance for recurrence can be expected if the model is trained by distinguishing the nAMD subtypes using a larger sample. Lastly, of the 421 patients who showed dry-up macula after three loading injections, only 269 patients (64%) with good follow-up examination were evaluated for recurrence, which may introduce bias. This could be attributed to variations in patient satisfaction or treatment response, which may affect patient compliance and thus create bias.

**Table 7:** Results of K-fold (K = 5) validation for the proposed model predicting on pre-injection images and all images immediately after each of the three injections.

K-Fold	Accuracy	Sensitivity	Specificity	F1score	Precision
Fold 1	0.5789	0.56	0.5938	0.5757	0.5789
Fold 2	0.5912	0.5448	0.6439	0.5810	0.5912
Fold 3	0.5737	0.5875	0.5	0.5223	0.5737
Fold 4	0.5965	0.6117	0.5304	0.5682	0.5965
Fold 5	0.5789	0.6897	0.4643	0.5725	0.5789

In conclusion, the AI model demonstrated remarkable ability for nAMD recurrence prediction using OCT images, surpassing the performance of experienced ophthalmologists. These results indicate that deep-learning models have the potential to aid in forecasting nAMD recurrence, ultimately enhancing patient outcomes and refining treatment approaches.

## Methods

### Data collection and labelling

We analyzed the medical records of the patients diagnosed with nAMD between January 2015 and June 2021 at the Kong Eye Hospital. Only treatment-naïve eyes with nAMD were enrolled and all the patients received three monthly injections of either ranibizumab or aflibercept. If both eyes were treated, only one eye was randomly selected. Exclusion criteria were extrafoveal nAMD; non-exudative AMD; more than a 6-week interval between three loading injections; prior treatment in the study eye with photodynamic therapy, subfoveal focal laser photocoagulation, or vitrectomy; anti-VEGF injection other than ranibizumab and aflibercept; macular degeneration, such as epiretinal membrane and macular hole; retinal vascular disease, such as retinal vein occlusion, retinal artery occlusion, and diabetic retinopathy; missing OCT examination; and cataract surgery within 3 months.

Age, sex, underlying diseases such as hypertension and diabetes, and history of ophthalmic surgery were recorded for all the patients. Visual acuity, intraocular pressure, and fundus examinations were performed, and neovascularization in the macula was confirmed using fluorescein angiography (FA) and indocyanine green angiography (ICGA). OCT was performed at each visit to determine the changes in the macula. FA was performed using the Heidelberg Retina Angiograph (HRA; Heidelberg Engineering, Heidelberg, Germany), and OCT was performed using the Heidelberg Spectralis (Heidelberg Engineering, Heidelberg, Germany).

OCT scans were performed prior to injection therapy and at every 4-week visit during injection therapy, and the treatment response was assessed using OCT scans performed 4 weeks after the third injection. The macular fluids were included the IRF, SRF, and PED. Dry macula was defined as the absence of IRF and SRF. Fluid under the retinal pigment epithelium was not considered for identifying dry macula unless the PED increased compared to that during the last visit. Regarding the treatment results, when all the IRF and SRF disappeared, the dry-up response was evaluated as good, and when the SRF and IRF remained and residual fluid was visible, the response was evaluated as poor. When only the PED remained and no other fluid was present, it was judged to be dry-up^9^. In cases of new macular hemorrhage or increased macular edema on OCT, a dry macula was not considered.

Upon OCT imaging, recurrence was determined if IRF, SRF, or subretinal hemorrhage (SRH) was observed, or PED was significantly increased. Recurrence was considered among only the patients who exhibited dry-up macula after three loading injections, if any signs of IRF, SRF, or SRH were observed, or if there was a significant increase in PED before the completion of follow-up (within 6 months after the initiation of the first treatment). However, if the dry-up state was maintained even 6 months after the initial injection treatment, it was classified as a non-recurrence group.

### Data preprocessing

**Figure 4:**
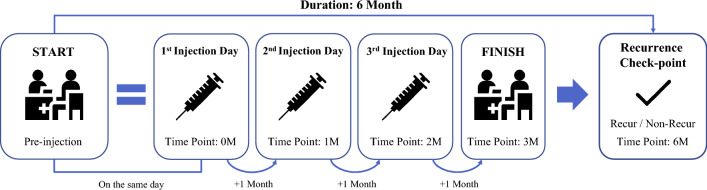
The flowchart of the three loading anti-vascular endothelial growth factor injections for patients with neovascular age-related macular degeneration. The process begins with capturing of the pre-injection optical coherence tomography (OCT) images on the first injection day, followed by monthly OCT image captures immediately following the injection. The recurrence is checked 6 months after the first injection day.

The flowchart illustrating the process of administering injections is depicted in Fig. 4. In our study, which aimed to predict the recurrence of SRF or IRF in the macula or increase of PED, 96 patients were excluded because they did not show dry-up macula after three injections. Additionally, 152 patients were excluded due to follow-up loss or missing follow-up around 6 months, making it difficult to evaluate the timing of recurrence around 4 months after the last injection. As a result, only 269 out of the initial 517 patients were included in the study. In addition, since we aimed to predict whether recurrence would occur within the next 3 months based on the follow-up time 1 month after the last injection, we used censoring statistical analysis^22^ to relabel the patient's recurrence; moreover, since some patients could not be tracked and data could not be recorded after three loading injections, data processing through censoring statistical analysis was considered necessary. Using the censoring statistic method, our dataset was divided into four cases: (1) recurrence within 4 months after the last injection, (2) recurrence after 6 months after the initial injection, (3) no recurrence after 6 months after the initial injection, and (4) no patient records after three injections. We relabeled (1) as having recurred, (2) and (3) as non-recurred and excluded (4) because we did not know whether recurrence occurred. Thus, we used the data of 269 relabeled patients as the final dataset by applying censoring statistical analysis to the 388 patients who completed the three loading injection treatments.

Moreover, because our research objective was to predict recurrence by examining the (1) pre-injection image only and (2) pre-injection image and all the images immediately after each of the three injections, we used 1076 SD-OCT images from 269 patients with pre-injection images and images after the 1st, 2nd, and 3rd injections.

We down sampled all the OCT images into a fixed-size image of 224 × 224 RGB for inputting deep neural network. We increased the various input images using data augmentation to build a robust model and avoid overfitting. The data augmentation process included (1) random horizontal image flips and (2) random rotations of up to 10° in the images. We performed data augmentation only during model training.

### Model architecture

To predict nAMD recurrence, we built a deep learning model based on DenseNet201^23^. As shown in Table 2, DenseNet201 demonstrated the best performance among other well-known CNN architectures, such as VGG-16^24^, Xception^25^, Inception-V3^26^, and ResNet-50^27^; thus, we selected DenseNet201 as the base feature extractor. DenseNet has the advantage of significantly reducing the number of parameters by encouraging reuse of the features^28^. Moreover, we confirmed that the deep-layer structure of Densenet201 captured the representations of the disease better than Densenet121 and Densenet169. To avoid overfitting and train the models faster, we applied transfer learning^29^ to learn the all models and to ensure fairness, the same input data were used in training model. Specifically, we initialized 200 layers of DenseNet201 with pre-trained weights using the large-scale Dataset, ImageNet^30^.

**Figure 5:**
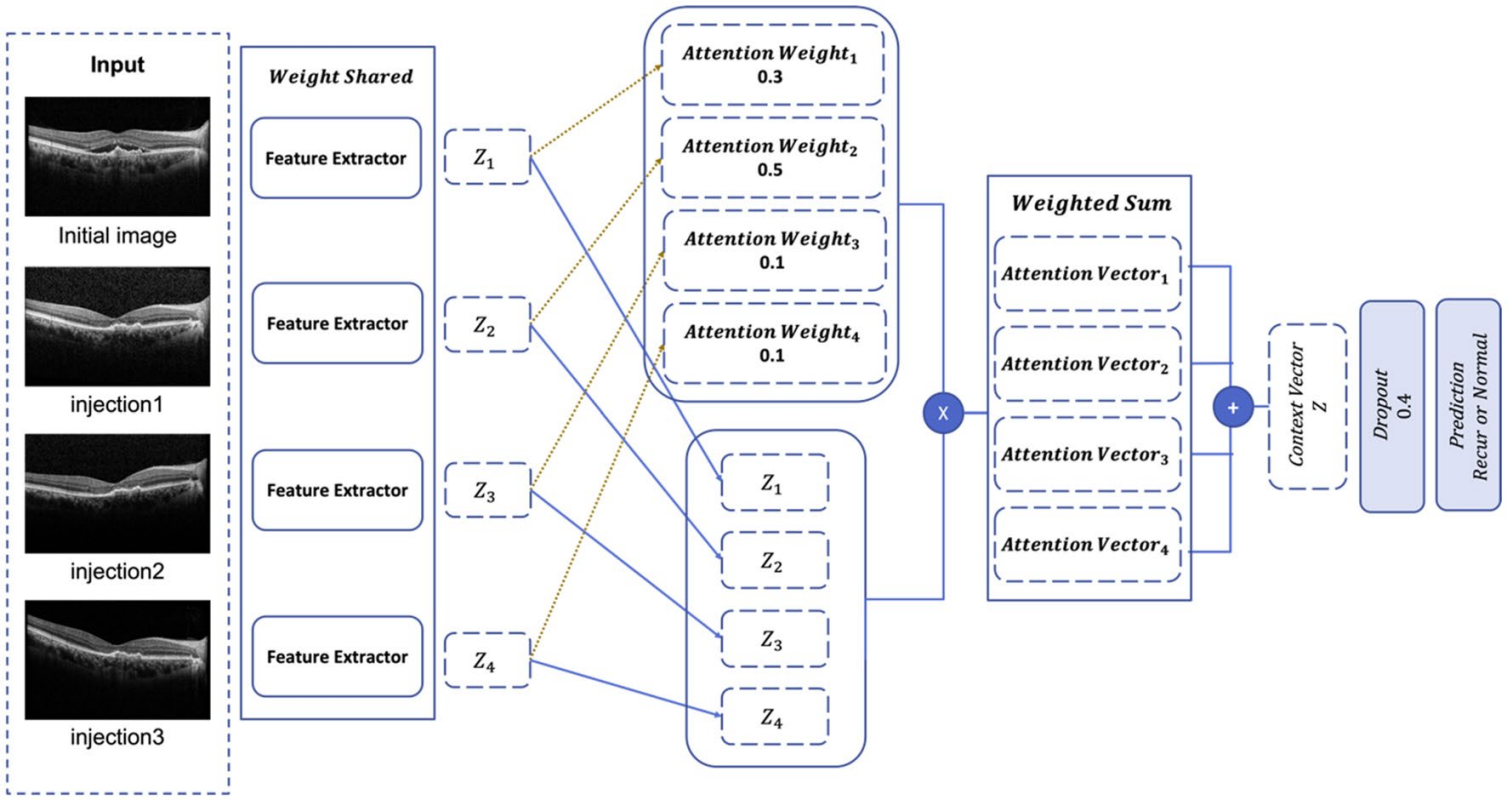
Overall architecture of the proposed model. The model is composed of an input layer, four feature extractors, an attention fusion layer, and a fully connected layer with dropouts and sigmoid activation function. The four feature extractors are based on DenseNet201 encoder, each with 200 pretrained convolutional neural network layers, and weight sharing application among them. The last fully connected layer predicts the likelihood of recurrence of the input cases within 6 months from the initial check point.

In addition, as shown in Fig. 5, we adopted a multi-instance model structure to simultaneously study multiple OCT images after monthly loading injections. To assess multiple input images, as shown in Table 3, both the LSTM^31^ and attention^32^ modules performed well in capturing sequential information. However, we selected the attention module as the final fusion method to calculate the attention score for each image and predict nAMD recurrence. While using the dropout layer to prevent overfitting, we added the traditional multilayered perceptron^33^ as a fully connected layer. Finally, we used the softmax activation function for the final output layers to predict the nAMD recurrence.

### Visual explanation using Grad-CAM

We applied Grad-CAM^34^ to provide a visual explanation of the decision-making process of the deep learning model. Grad-CAM highlights the important regions on OCT images for predicting nAMD recurrence through gradient-based localization. Based on the gradient of the feature maps of each convolution layer, we created a heat map representing the part used in the prediction process of the model.

### Experimental setup

We performed LOOCV^35^ to train both the baseline and proposed models. Thus, LOOCV is an effective validation method for small data sizes^21^. For applying LOOCV to our training process, we retained only one data sample for model testing and used the remaining dataset for training. This process was repeated 269 times, indicating the number of patients, with each observation being excluded once as validation data. Although LOOCV is a well-known effective method for evaluating small data sizes, it is also known for its vulnerability to overfitting compared to K-fold cross-validation^36^. To manage overfitting, we employed dropouts and implemented early stopping with a patience of 7. To perform LOOCV for all the datasets, we divided the dataset according to the patient ID to prevent the OCT images of the same patient from being mixed in the training, test, and validation sets. In addition, we employed the same LOOCV to train all the models, and each model was evaluated using the average performance for all the LOOCV results. To ensure fairness across all models, we established a uniform parameter standard that configured the batch size, epoch, and dropout rate as 64, 100, and 0.4, respectively. Additionally, we utilized the Adam^37^ optimization with a learning rate of 0.001 for all models, including the proposed model.

For the general experiment, 100 samples were randomly selected from 269 patients for a performance comparison with the ophthalmologists. To ensure a fair experiment, these 100 samples were not used in the model training process. We provided (1) a single image of the initial state, before the loading injections, and (2) three post-injection images immediately after the loading injection of these 100 patients to six ophthalmologists, including two ophthalmology residents (1 and 3 years of experience, respectively), three retinal fellows (2 years of experience as a retina specialist), and one retinal specialist (more than 10 years of clinical experience). Note that, during the experiment involving the presentation of (1) a single image of the initial state before the loading injections and (2) three post-injection images immediately after the loading injection to six ophthalmologists, only (1) was shown while (2) was intentionally withheld. To analyze the ophthalmologists' individual and common perspectives, we analyzed each ophthalmologist's prediction results for 100 patients, consisting of 52 recurrence cases and 48 non-recurrence cases. Simultaneously, we calculated the average decision-making and probability of recurrence by all the ophthalmologists for comparison with the softmax value of the proposed model. If all six ophthalmologists predicted recurrence for the sample, it was calculated as 1, and if all six predicted no recurrence, it was calculated as 0. Statistical analysis was conducted between the average prediction rate of the six ophthalmologists and the softmax value of the proposed model to determine the consistency and relevance of the decision-making process.

### Statistical analysis

We applied Fleiss' kappa coefficients to calculate the level of agreement between the multiple rates, including those of all the ophthalmologists and the proposed model. To compute this statistic, we used the Statsmodels module, a well-known Python package for statistical analysis.

### Ethical approval

This study adhered to the principles of the Declaration of Helsinki and was approved by the Institutional Review Board of Kong Eye Hospital (KIRB-202202-HR-001-01), which waived the requirement for obtaining informed consent because this was a retrospective observational study of medical records and was retrospectively registered.

## Data Availability

The data are not available for public access owing to patient privacy concerns; however, data are available from the corresponding author upon reasonable request.
